# Isolation and Genetic Characteristics of Human Genotype 1 Japanese Encephalitis Virus, China, 2009

**DOI:** 10.1371/journal.pone.0016418

**Published:** 2011-01-25

**Authors:** Jiu-Song Zhang, Qiu-Min Zhao, Xiao-Fang Guo, Shu-Qing Zuo, Jing-Xia Cheng, Na Jia, Chao Wu, Pei-Fang Dai, Jun-Ying Zhao

**Affiliations:** 1 State Key Laboratory of Pathogen and Biosecurity, Beijing Institute of Microbiology and Epidemiology, Beijing, People's Republic of China; 2 Yunnan Institute of Parasitic Diseases, Simao, People's Republic of China; 3 Shanxi Center for Disease Control and Prevention, Taiyuan, People's Republic of China; Duke-National University of Singapore Graduate Medical School, Singapore

## Abstract

**Background:**

Several studies have shown that the predominant genotype of Chinese Japanese encephalitis virus (JEV) is evolving from genotype 3 to genotype 1. However, in recent years, almost all genotype 1 isolates were from mosquitoes, and genotype 1 has been less associated with human disease than genotype 3. This study reports the isolation of human genotype 1 JEV and its genetic characteristics to provide additional insights into human JE pathogens that are currently circulating in China.

**Methods and Results:**

In 2009, 31 cerebrospinal fluid samples were collected from patients living in Yunnan and Shanxi provinces and were used to inoculate *Aedes albopictus* C6/36 cells for virus isolation. The JEV strains were identified using immunofluorescent assays and the reverse transcription-polymerase chain reaction. Phylogenetic analyses based on the partial capsid/pre-membrane and full envelope (E) sequences were performed using Clustalx 1.8 software. Three JEV isolates were obtained from a 4-year-old girl and a 2-year-old boy living in Yunnan and an 82-year-old woman in Shanxi. The boy had been immunized with one dose of JE live attenuated vaccine. New isolates were grouped into genotype 1. Amino acid sequence for the viral E protein indicated 95% to 100% identity with each other and with other JEV strains. When compared with a consensus sequence of E protein, two amino acid substitutions were found: Ser^E-123^-Asn in the two Yunnan isolates and Lys^E-166^-Arg in the Shanxi isolate.

**Conclusions:**

Our findings indicate that the genotype 1 of JEV is causing human infections in China. Our observation of a previously vaccinated boy developing JE from genotype 1 virus infection also calls for more detailed studies, both in vitro and in vivo neutralization tests as well as active surveillance, to examine the possibility of a lack of complete protection conferred by the live attenuated JE vaccine against genotype 1 virus.

## Introduction

Japanese encephalitis (JE) is prevalent in eastern and southern Asia. An estimated 3 billion persons live in areas where JE is endemic and the annual incidence of the disease in these areas is 30,000–50,000 cases [1], [2]. Approximately 25%–30% of cases are fatal, and 50% result in irreversible neuropsychiatric sequelae. The disease primarily affects children and adolescents. In China, JE is the most important viral encephalitis and is one of four currently circulating arbovirus diseases [3]. Epidemic JE activity has been detected in the last 60 years in most provinces of mainland China, except for Xinjiang Uygur Autonomous, Tibet and Qinghai Province. Although nationwide immunization has dramatically reduced the disease incidence, there are still 8,000–10,000 cases reported annually, contributing significantly to the global disease burden [4].

JE virus (JEV), a member of the genus *Flavivirus* of the family *Flaviviridae*, is maintained in nature through a transmission cycle involving the *Culex* species mosquitoes, pigs, bats, and water birds [5]. The viral genome is a positive-sense, single-stranded RNA that contains a single open reading frame encoding three structural proteins designated capsid (C), pre-membrane/membrane (prM/M) and envelope (E), and seven non-structural proteins, NS1, NS2A, NS2B, NS3, NS4A, NS4B and NS5 [6]. Partial sequence analysis of the C/prM gene indicates that JEV strains can be classified into four genotypes [7], [8].

In China, genotype 3 JEV was the predominant genotype before 2001, but since then, genotype 1 isolates have been frequently detected [9]. Moreover, an outbreak of JE occurred in 2006 in the Chinese province of Shanxi; the outbreak consisted of 66 cases with a 28.8% fatality rate, and both genotypes 1 and 3 were detected in the patients [10]. These observations imply that the predominant genotype of Chinese JEV is changing from genotype 3 to genotype 1 or that the co-circulation of the two genotypes is becoming common. However, most of the genotype 1 JEV isolates in recent years were from mosquito pools; furthermore, this genotype been less associated with human disease than genotype 3 [11], [12]. The general lack of knowledge regarding the prevalence and severity of genotype 1 JEV led us to investigate the predominant JEV genotype within a group of JE patients in China. In the present study, we report the isolation of genotype 1 JEV in humans and the genetic characteristics of the E protein of this genotype.

## Materials and Methods

### Ethics statement

The study was performed after consultation with the patients or their guardians and after the receipt of written consent. The study-related information was used anonymously. The Institutional Review Board of the Beijing Institute of Microbiology and Epidemiology approved the research involving human materials. The animal experiments were approved by the Animal Subjects Research Review Board of the Beijing Institute of Microbiology and Epidemiology and were conducted according to the institution's guidelines for animal husbandry.

### Sample collection

From June to August 2009, a total of 78 patients who had a clinical diagnosis of viral encephalitis were hospitalized: 19 from Luxi City and 30 from Banna in Yunnan Province; 5 from Linyi County and 24 from Taiyuan City in Shanxi Province. Cerebrospinal fluid (CSF) samples were collected from all 78 patients. Thirty-five cases were determined to be JE with positive findings of IgM antibodies in the CSF samples. The 31 CSF samples collected within five days of illness onset were selected for virus isolation. Yunnan Province, located in southern China, has a tropical to subtropical climate and a high JE incidence (>1/100,000 people). Shanxi Province, located in middle China, has a JE incidence rate of 0.1–0.5/100,000 people [13]. Linyi County was a main epidemic area in the 2006 JE outbreak mentioned above. All samples were stored in liquid nitrogen until tested.

### Viral isolation and identification

The CSF specimens were diluted at a 1∶5 ratio with RPMI-1640 medium containing 2% fetal calf serum and cultured with *Aedes albopictus* C6/36 cells for 7–10 days. Cultures were examined daily for evidence of a virally-induced cytopathic effect (CPE). Infected-cells that showed CPE were harvested and identified as JEV-positive by an immunofluorescent assay (IFA) using JEV-polyclonal antisera. The culture supernatant was filtered with a 0.22-µm filter and was then used to inoculate suckling mice intracebrally for an evaluation of the pathogenicity of the isolated virus. Cultures without CPE were blind-passaged 3 times.

Total RNA was extracted from the infected C6/36 cell culture using Trizol and reverse-transcribed with random hexamers to obtain first-strand cDNA. For primary molecular identification, PCR was performed with a pair of JEV-specific primers in the NS1 region: 5′-GTGCCATTGACATCACAAG-3′ and 5′-TGTCTCAGGTCCATCTACG-3′. In JEV-positive samples, partial C/prM (675 bp) and the full E genes of JEV were amplified as described by Wang et al. [9]. PCR products were directly sequenced using an automated DNA sequencer (ABI PRISM 373, Perkin-Elmer, USA). The DNA was sequenced using both forward and reverse primers to verify the sequences. The sequences were subjected to a BLAST search.

### Phylogenetic analysis

A total of 50 JEV sequences were used for phylogenetic analyses, including 39 representative Chinese sequences. Multiple sequence alignments were carried out using Clustalx 1.8 software [14]. Phylogenetic trees were generated using the Bayesian Metropolis-Hastings Markov Chain Monte Carlo (MCMC) tree-sampling methods implemented by Mr. Bayes 3.1 software. We used the GTR model as the evolutionary model with gamma-distributed rate variation across sites and a proportion of invariable sites [15]. The run was stopped when the standard deviation of split frequencies was below 0.01. A West Nile virus (WNV-NY99) sequence was used as an out-group control.

## Results

### Case summaries

The basic characteristics of the patients are summarized in Table 1. The patients from Luxi City and Taiyuan City were almost all children or adolescents, with an average age of 8–9 years. In contrast, the patients from Linyi County were all adults; of the five cases, three were >60 years old, with an average age of 55 years.

**Table 1:** Basic characteristics of the patients participating in this study.

Area	Number	Male/Female	Average age (range) (yrs)
Luxi City, Yunnan	20	16/4	9 (2–23)
Linyi County, Shanxi	5	3/2	55 (29–82)
Taiyuan City, Shanxi	6	3/3	8 (4–10)

Viral isolates were obtained from a 4-year-old girl, a 2-year-old boy and an 82-year-old woman, designated as LX10P-09, LX29P-09 and LY5P-09, respectively. The positive isolation rate was approximately 10%. Both the 4-year old girl and 2-year old boy lived in Luxi City, Yunnan Province. The girl visited a local hospital on July 7 with symptoms of fever, headache, vomiting, convulsion and drowsiness. Neck stiffness was also noticed. The boy visited the same hospital on June 30 with symptoms of fever, and vomiting. He received one dose of live-attenuated JE vaccination 15 months prior to presentation. The woman patient, residing in Linyi County, was hospitalized on August 13 for fever, vomiting, drowsiness and convulsion. According to the case definition by the Ministry of Health of the People's Republic of China, the girl, woman and boy patients were recognized as having clinically severe, very severe and mild JE, respectively. Their acute-phase CSF samples were collected at day 1–2 after the onset of illness. The JEV-specific immunoglobulin M (IgM) antibodies from the CSF, measured by IgM antibody-capture enzyme-linked immunosorbent assay (MAC-ELISA), were positive in the girl patient but negative in two other cases (Table 2).

**Table 2:** Summarized data of the patients from whom the viruses were isolated.

	Gender	Age(yrs)	Residence	JEvaccination	Clinical type	JEV-IgMin CSF	Isolate
Patient 1	female	4	Luxi City	unknown	severe	positive	LX10P-09
Patient 2	male	2	Luxi City	yes	mild	negative	LX29P-09
Patient 3	female	82	Linyi County	no	very severe	negative	LY5P-09

### Viral isolation

The virus isolates induced CPE in first-passage C6/36 cells on day 5–6 after inoculation. Cell shrinking, shedding, fusion or cytolysis characterized the CPE. Cells infected by the viral isolates all reacted with JEV-polyclonal antisera in IFA. Suckling mice that were inoculated intracerebrally with the isolates all became ill and died within 48–96 h. The three viral isolates studied were positive for JEV RNA; the JEV-positive samples were identified by RT-PCR using JEV-specific primers in NS1, C/prM and E regions and were matched to JEV using a BLAST search. The sequences of partial C/prM and full E genes were deposited intothe GenBank database with accession numbers GU371870, HM204528 (LX10P-09), GU371871, HM204529 (LX29P-09) and GU371872, HM204530 (LY5P-09).

### Genetic characteristics of the isolates

**Figure 1:**
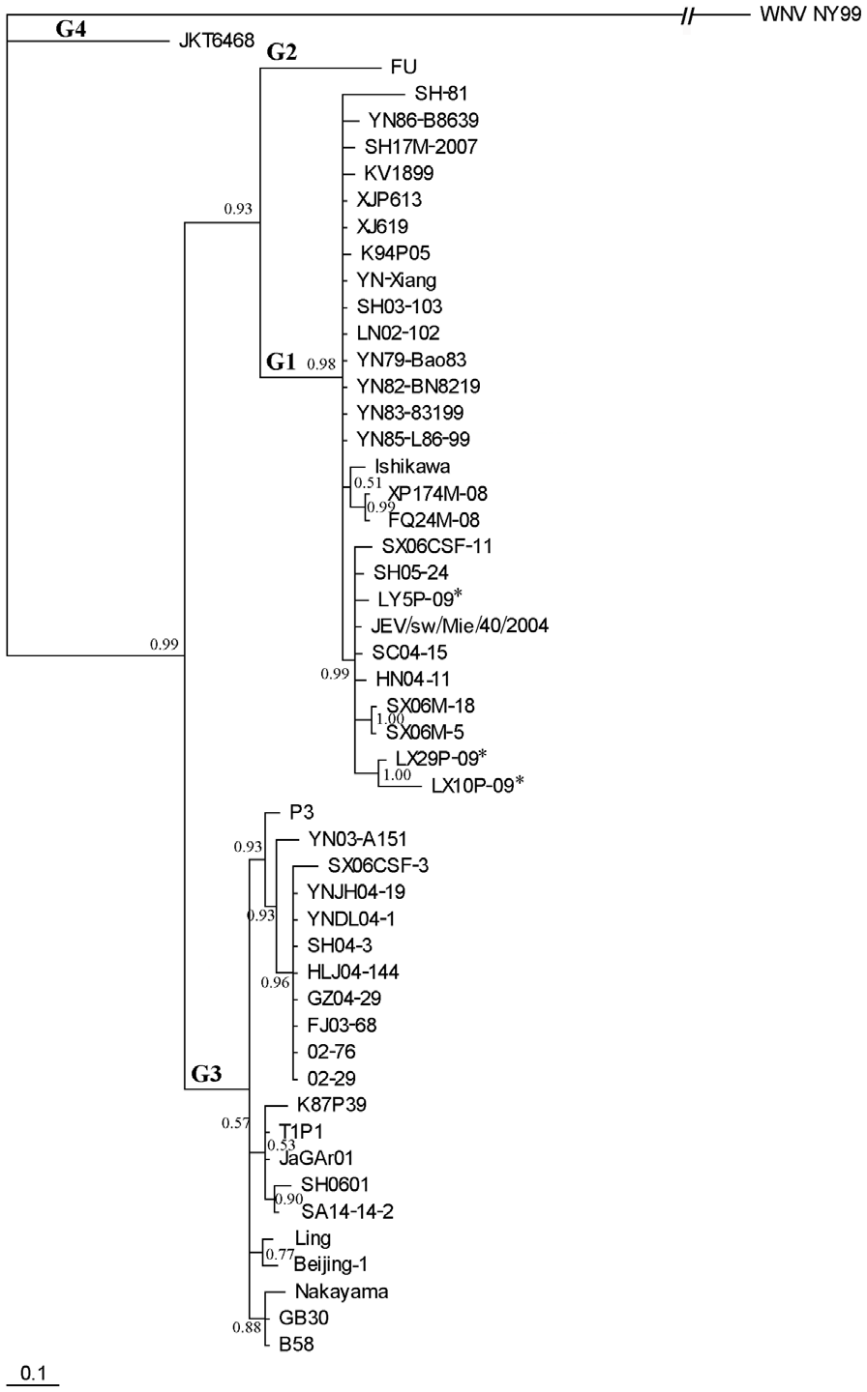
Phylogenetic tree based on the C/prM gene of the representative JEV strains. The details of the strains, except SX06CSF-3, SX06CSF-11 (China, Shanxi, human CSF, 2006), and SX06M-5, SX06M-18 (China, Shanxi, mosquito, 2006), are presented in Figure 2. WNV NY99 (NC_009942) was used as an out-group. JEV Genotypes 1–4 are expressed as G1–G4, respectively. An asterisk is used to denote the three isolates used in this study.

A 240 bp fragment of the 675 bp C/prM gene was utilized for multiple sequence alignments and phylogenetic analyses. This region was used to determine the genotype of JEV. Except for Indonesia JKT6468 which was recognized as genotype 4, 49 sequences were grouped into two distinct phylogenetic groups with high bootstrap support (Fig. 1). The Australian FU recognized as JEV genotype 2 was classified into its own clade. Another cluster in the same major group consisted of 27 closely related sequences and branched into many minor clades. The three isolates from this study grouped into the same clade with 7 other strains isolated within the past five years including one Japanese and 6 Chinese sequences: SX06CSF-11, SX06M-5 and SX06M-18 from Shanxi, HN04-11 from Henan, SC04-15 from Sichuan, SH05-24 from Shanghai City, and JEV/sw/Mie/40/2004 from Japan. The new isolates were classified as JEV genotype 1.

**Figure 2:**
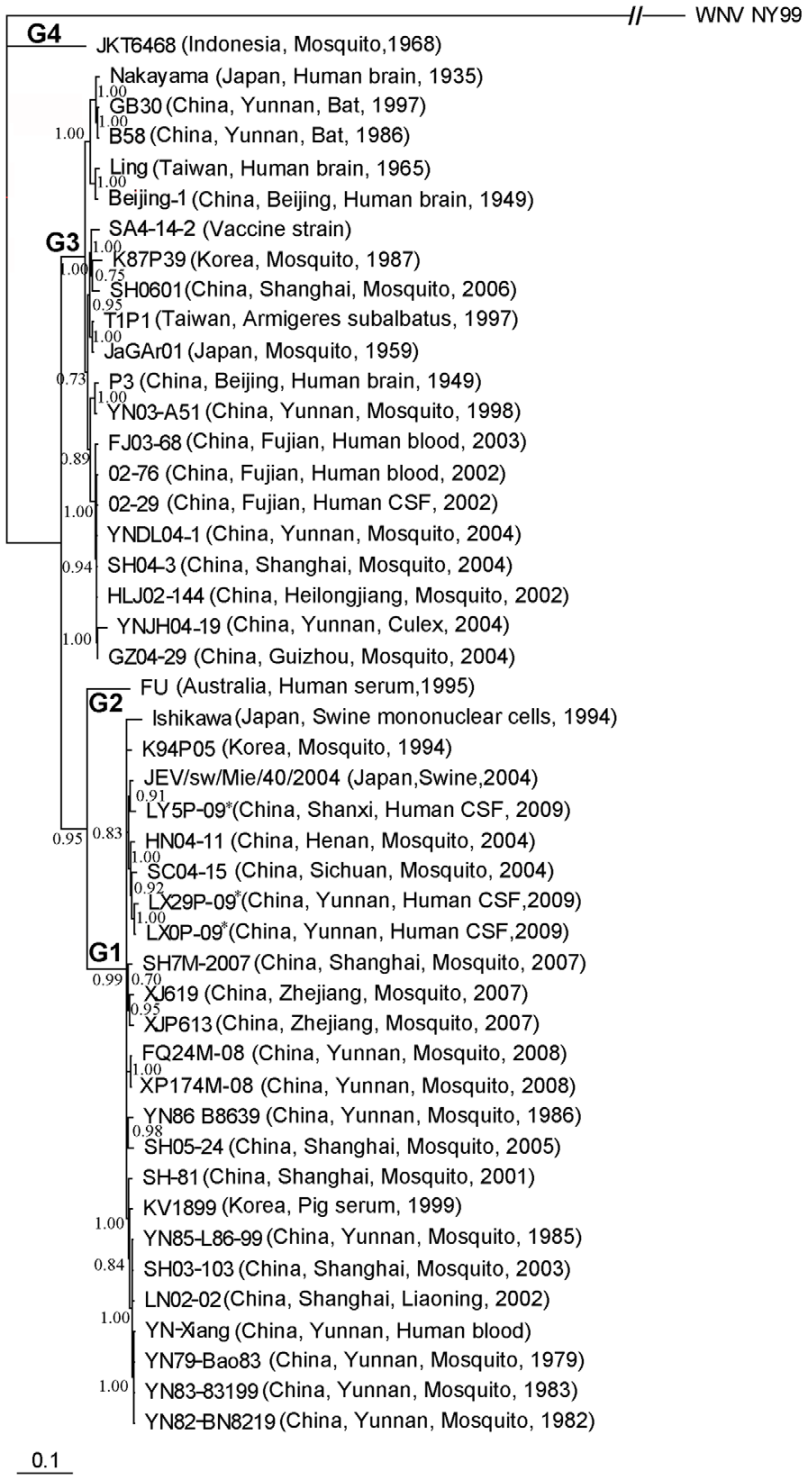
Phylogenetic tree based on the full E gene of the representative JEV strains. WNV NY99 (NC_009942) was used as an out-group. JEV Genotypes 1–4 are expressed as G1–G4, respectively. An asterisk is used to denote the three isolates used in this study.

A phylogenetic tree according to the full E sequence revealed a similar trend with the tree based on the C/prM region (Fig. 2). The three new isolates were genetically related to each other and had high similarity (99.6%–100%) with respect to the deduced amino acid sequence of E protein. When compared to other JEV strains used in this study, they had 81.4%–98.9% identity at the nucleotide level and 95.2%–100% identity at the amino acid level. There was 97.0% homology with the Chinese vaccine strain SA14-14-2 (attenuated SA14). Table 3 summarizes the amino acid differences in the E protein sequence among the new isolates and SA14-14-2 strain. There were 16 substitutions in the 500 amino acid residues of E protein. When compared with the consensus sequence from 46 E proteins, two amino acid residues were different: Ser^E-123^→Asn in the two Yunnan isolates and Lys^E-166^→Arg in the Shanxi isolate.

**Table 3:** Comparison of amino acid differences in E proteins in the new isolates and the vaccine strain.

Position	SA14	SA14-14-2	LX10P-09	LX29P-09	LY5P-09
E107	Leu	Phe	Leu	Leu	Leu
E123	Ser	Ser	Asn	Asn	Ser
E129	Thr	Thr	Met	Met	Ser
E138	Glu	Lys	Glu	Glu	Glu
E166	Lys	Lys	Lys	Lys	Arg
E176	Ile	Val	Ile	Ile	Ile
E177	Thr	Ala	Thr	Thr	Thr
E222	Ala	Ala	Ser	Ser	Ser
E244	Glu	Gly	Glu	Glu	Glu
E264	Gln	His	Gln	Gln	Gln
E279	Lys	Met	Lys	Lys	Lys
E315	Ala	Val	Ala	Ala	Ala
E327	Ser	Ser	Thr	Thr	Thr
E366	Ala	Ala	Ser	Ser	Ala
E439	Lys	Arg	Lys	Lys	Lys
E447	Gly	Asp	Gly	Gly	Gly

## Discussion

In recent years, JEV genotype 1 has been frequently isolated at different geographical areas in mainland China including Liaoning (2002), Sichuan (2004), Henan (2004), Shandong (2009), Guizhou (2010) and Shanghai (2001, 2003, 2005, 2007) [9], [12], [16], [17]. However, these isolates were primarily from mosquito pools or porcine samples. Since the early-1990s, human JEV isolation was neglected in China, and the isolation of genotype 1 JEV in human was rarely reported [12]. Using RT-PCR, Wang et al. [10] detected nucleotide segments of JEV from human CSF samples acquired in 2006, and four of six cases were classified as genotype 1. Further evidence of genotype 1 JEV circulation was found with the analysis of meningitis patients in Japan [11]. In the present study, three JEV isolates in patients from Yunnan and Shanxi were classified into genotype 1. It would be interesting to determine whether genotype 1 has become the predominant genotype among human JE cases in China. To confirm this finding, additional human isolates will be necessary.

Yunnan Province is a region with a high incidence of JE [13]. Controlling the JE incidence in Yunnan is crucial to decreasing the disease burden in China. A limited survey showed that more than 10% of mosquito pools were positive for JEV in 2007 and 2008 (our unpublished data), indicating that JEV is still prevalent in Yunnan. We obtained JEV strains from two ill patients in this area: a young boy and girl. Unexpectedly, the boy patient had been immunized with one dose of JE live attenuated vaccine, which is prepared based on genotype 3 of JEV and used commonly for human vaccination in China [18]. It is unknown whether the boy's infection results from a lack of immune response to or protective immunity against circulating JEV. To answer this question, neutralization tests on acute and convalescent serum samples should be conducted. Unfortunately, we could not obtain serum samples from the patient. The boy patient was a clinically mild case without reduced level of consciousness, neck stiffness or convulsion. Interestingly, we found that another patient, a 4-year-old boy, had also previously received one dose of a JE vaccine, and he was also confirmed as having JE and positive JEV-IgM in his CSF, but revealed mild clinical manifestations. It is tempting to speculate that JE vaccination might alleviate symptoms of the disease, even though it may not prevent infection by JEV.

Although Shanxi Province was not at high risk for a JE epidemic, an outbreak of viral encephalitis occurred in Yuncheng, Shanxi Province, in 2006. Generally, JE affects children, but during the 2006 Shanxi outbreak, a higher adult incidence was observed; in fact, approximately 95% of the disease-related deaths occurred in patients over 50 years old [10]. We identified five patients with viral encephalitis in Linyi County, Yuncheng in 2009, and all patients were adults with an average age of 55 years. This observation suggested that adults, especially the elderly, in this region are at higher risk of JE; it is reasonable, therefore, that public health officers should recommend JE vaccination to this population. Overall, there is less information about JEV strains from Shanxi Province. We isolated JEV from an 82-year-old patient, and to our knowledge, it is the first isolate from an elderly patient. Analysis of a larger number of isolates along with that reported in this study would be needed to determine the transmission pattern of JE in various Shanxi Province.

The E protein of flaviviruses, including JEV, plays an important role in immunogenicity, tissue tropism, cell fusion and infection, and virus maturation [19]. Several studies have suggested that some amino acid substitutions in the E protein may be related to the virulence of JEV. Through a comparison of the amino acid sequences between wild-type SA14 and its attenuated strains, Ni et al. [20] found that mutations in E138 (Glu→Lys), E176 (Ile→Val), E315 (Ala→Val) and E439 (Lys→Arg) may contribute to the attenuation of neuroinvasion and neurovirulence of JEV. Our three isolates were identical to the wild SA14 strain in these sites (Table 3). Recently, Tajima et al. [21] demonstrated that a single mutation in E123 (Ser to Arg) resulted in a significantly increased viral growth rate in mouse neuroblastoma cells; The two Yunnan isolates showed a mutation in E123 (Ser to Asn), but the biological significance of this mutation is unknown.

Previous reports showed that the antigenic epitopes of JEV are located within certain regions of the E protein; for example, residues 331 and 332 were shown to be part of a neutralizing epitope, and residues 60–68 are recognized by cytotoxic T lymphocytes [22]–[24]. The new isolates did not have any changes compared to the JE vaccine strain SA14-14-2 in these regions. However, considering the case of the boy who has been vaccinated with the live attenuated JE vaccine but developed mild JE, protective immunity to JEV may be also related with the mutation in other viral proteins besides E protein. Several studies have demonstrated that the NS1 protein induced protective immune responses against lethal JEV challenge. When immunized with the plasmid expressing NS1, 90% of the mice survived after a lethal JEV challenge, whereas when injected with the plasmid expressing prM plus E, 70% of the immunized mice survived after a lethal challenge [25]. Pigs vaccinated with recombinant pseudorabies virus expressing NS1 protein of JEV SA14-14-2 strain could develop a good humoral and cellular immune response against JEV [26]. A recent report indicated a high immunogenicity and significant protection of *E. coli* synthesized NS1 protein to JEV in the mice [27]. In addition, the mutations in other E regions of new isolated strains, which were not reported to be associated with protective immunity, should be concerned.

Our findings indicate that the genotype 1 of JEV is causing human infections in China. Our observation of a previously vaccinated boy developing JE from genotype 1 virus infection also calls for more detailed studies, both in vitro and in vivo neutralization tests as well as active surveillance, to examine the possibility of a lack of complete protection conferred by the live attenuated JE vaccine against genotype 1 virus.
